# The Malignancy of Melanotic Sarcoma

**Published:** 1910-03-19

**Authors:** 


### THE MALIGNANCY OF MELANOTIC SARCOMA.

As a general rule a pigmented malignant growth of the kind commonly called melanotic sarcoma, which some modern pathologists are inclined to class rather with the carcinomata, is of the gravest prognostic import. This disease is rightly regarded as of unusual virulence, and consequently as requiring early and radical operation even beyond other cancers. It has to be remembered, too, that since it most frequently arises on some surface where it is easily observed, and since its peculiarity of black pigmentation is so characteristic, melanotic sarcoma is, as a rule, easily diagnosed in a comparatively early stage, so that its evil reputation is the reverse of what might be expected.

While this sums up the usual experience of surgeons and practitioners who have to deal with cases of this disastrous lesion, there are reported from time to time cases in which the malignancy of the tumour is only slight, or is latent, or even is apparently lost altogether. A very remarkable instance of the last-mentioned peculiarity was given in detail in these pages two years ago (The Hospital, March 14, 1908, p. 629), in the course of an article on the Spontaneous Disappearance of Malignant Growths; the full account of this extraordinary case when it was originally reported by Sir Wm. Bennett was published 11 years since.* Yet another example of progressive loss of malignancy in a melanotic sarcoma, though not to so remarkable an extent, has lately been published by Mr. C. H. Leaf.† In this case the patient, a woman, suffered since childhood from a wart on the sole of the foot, which for some years prior to her first admission to hospital increased in size, ulcerated and caused pain. In 1903 the patient discovered a swelling on the front of the knee, which was removed by a doctor and pronounced to be a sarcoma. In 1904 the woman came under the care of the surgeon who now reports the case, and at that time she had a small fungating growth close to the bases of the second, third, and fourth toes. There was an enlarged gland over the saphenous opening, and also two nodules on the outer side of the thigh. The patient refused to have an amputation or any other radical measure performed, so only the growths were removed, together with the glands in the saphenous opening; all of these showed the typical structure of a melanotic sarcoma. In 1909 this woman reappeared in a very fair state of health, but with two recurrent nodules of growth near the scar on the foot, and a pigmented mass the size of a walnut on the inner aspect of the leg, six inches below the knee. Another smaller nodule was found in the calf, and over the upper part of the popliteal space was a tumour as large as an orange, which was adherent to deeper structures but not to the bone. An enlarged gland was found over the saphenous opening. In October 1909 the popliteal mass and the saphenous glands were removed. Later the various smaller masses were excised, and the patient went home in November. The microscopical examination of the tumours showed them to be polymorphic-celled sarcomata with areas of pigmentation. Here again a striking similarity with Sir Wm. Bennett's case exists, for in that also the original growth was deeply pigmented, and the successive recurrences were gradually less and less so. Whether, as in that case, this patient is also in process of spontaneous cure is a question which cannot now be answered. It would have been interesting to have watched the case to ascertain this, but, of course, quite unjustifiable in the interests of the patient, which have beyond doubt best been served by the surgical procedures already adopted. It may be hoped that this case will be followed up and again reported on at intervals of a year or so.

* *The Lancet*, January 7, 1899.

† *West London Medical Journal*, January 1910, p. 63.

